# MicroRNA-506 as a tumor suppressor in anaplastic thyroid carcinoma by regulation of WNT and NOTCH signaling pathways

**DOI:** 10.22038/IJBMS.2023.69174.15069

**Published:** 2023

**Authors:** Zahra Nasrpour Navaei, Negin Taghehchian, Amir Sadra Zangouei, Mohammad Reza Abbaszadegan, Meysam Moghbeli

**Affiliations:** 1 Department of Medical Genetics and Molecular Medicine, Faculty of Medicine, Mashhad University of Medical Sciences, Mashhad, Iran; 2 Medical Genetics Research Center, Mashhad University of Medical Sciences, Mashhad, Iran; 3 Student Research Committee, Faculty of Medicine, Mashhad University of Medical Sciences, Mashhad, Iran

**Keywords:** Anaplastic thyroid – carcinoma, Chemo resistance, EMT, miR-506, NOTCH, WNT

## Abstract

**Objective(s):**

Anaplastic thyroid carcinoma (ATC) is an aggressive thyroid tumor type that has a poor prognosis due to its high therapeutic resistance. Since ATC accounts for half of thyroid cancer-related deaths, it is required to introduce novel therapeutic targets to increase survival in ATC patients. WNT and NOTCH signaling pathways are the pivotal regulators of cell proliferation and migration that can be regulated by microRNAs. We assessed the role of miR-506 in the regulation of cell migration, apoptosis, and drug resistance via NOTCH and WNT pathways in ATC cells.

**Materials and Methods:**

The levels of miR-506 expressions were assessed in ATC cells and tissues. The levels of NOTCH, WNT, and EMT-related gene expressions were also assessed in miR-506 ectopic expressed cells compared with controls. Cell migration and drug resistance were also evaluated to assess the role of miR-506 in the regulation of ATC aggressiveness.

**Results:**

There were significant miR-506 down-regulations in ATC cells and clinical samples compared with normal cells and margins. MiR-506 suppressed NOTCH and WNT signaling pathways through LEF1, DVL, FZD1, HEY2, HES5, and HEY2 down-regulations, and APC and GSK3b up-regulations. MiR-506 significantly inhibited ATC cell migration and EMT (*P*=0.028). Moreover, miR-506 significantly increased Cisplatin (*P*=0.004), Paclitaxel (*P*<0.0001), and Doxorubicin (*P*=0.0014) sensitivities in ATC cells.

**Conclusion:**

MiR-506 regulated EMT, cell migration, and chemoresistance through regulation of WNT and NOTCH signaling pathways in ATC cells. Therefore, after confirmation with animal studies, it can be introduced as an efficient novel therapeutic factor for ATC tumors.

## Introduction

Thyroid cancer (TCa) is the most common malignancy of the endocrine system (1). It is considered the eighth most frequently diagnosed cancer globally (2), and its worldwide incidence has been rising during the past decades (3). TCa is histologically categorized into papillary, follicular, medullary, and anaplastic thyroid carcinoma (ATC) (4). There are various therapeutic options for TCa patients including surgical resection, chemotherapy, hormone suppression therapy, tyrosine kinase inhibitors, and anti-angiogenic agents (5). Papillary thyroid carcinoma (PTC) involves up to 85%, while other thyroid tumor types account for 5% of all TCa (6). ATC is an aggressive thyroid tumor type that has a poor prognosis due to its high therapeutic resistance. Although ATC is a rare type of thyroid tumor that accounts for 1–2% of cases, it is responsible for up to 50% of thyroid cancer-related deaths (7). Therefore, it is required to introduce novel therapeutic modalities to increase patient survival in ATC patients. Epithelial-mesenchymal transition (EMT) is a cellular process highlighted by the loss of epithelial features and acquisition of mesenchymal properties that promotes cell migration potentials (8). It has critical roles in different physiological and pathophysiological processes including embryonic development, organogenesis, and tumor metastasis (9). EMT also confers chemoresistance via activation of alternative survival pathways, apoptosis suppression, and up-regulation of drug efflux pumps (10, 11). Wnt and NOTCH signaling pathways are the main regulators of the EMT process (12). WNT comprises a diverse family of glycoproteins that serve as ligands for Frizzled family of cell surface receptors which regulate cellular differentiation, proliferation, and tissue homeostasis (13). Deregulation of WNT is also associated with tumor progression (14). It has also been shown that the WNT pathway promotes cancer invasiveness by inducing and stabilizing various EMT activators (15). NOTCH signaling is a developmental pathway that is implicated in the regulation of cell proliferation, differentiation, and apoptosis (16). Considering the fundamental role of NOTCH signaling in cellular differentiation, there is no surprise that aberrancies in this pathway cause neoplastic transformation (17). NOTCH signaling also regulates the EMT process via its interactions with growth and transcription factors involved in EMT such as Slug, Snail, PDGF, FGF, and TGF-β (18). MicroRNAs (miRNAs) are endogenous short non-coding RNAs that bind to the 3′ un-translated region (3-UTR) of their target mRNAs and serve as critical post-transcriptional regulators (19). MiRNAs are implicated in the modulation of key biological mechanisms such as cell proliferation, growth, and apoptosis (20). Based on their target mRNAs, miRNAs have a context-dependent function and can act both as tumor suppressors or oncogenes (21), and their aberrant expression has been correlated with various cancers (22). Different components of Wnt and NOTCH signaling pathways are regulated by miRNAs during embryogenesis and tumorigenesis (23, 24). While some studies introduce miR-506 as a tumor suppressor agent (25, 26), miR-506 exhibits oncogenic function in some other cases (27, 28). In the present study for the first time, we assessed the probable role of miR-506 in ATC cell migration, EMT, and drug response.

## Materials and Methods

***Tissues***

Five paraffin-embedded ATC tissues and their corresponding normal margins were collected from Imam Reza Hospital of Mashhad University of Medical Sciences. All the patients filled out informed consent forms that were confirmed by the ethics committee. 

***Cell culture and transfection***

The human thyroid cancer cell line 8305c was obtained from (Pasteur Institute, Iran) and cultured in DMEM-high glucose medium containing 10% fetal bovine serum, 100 U/ml penicillin, and 100 lg/ml streptomycin. MiR-506-pcDNA.3 vector was designed and used for miR-506 ectopic expression in the 8305c cell line. The cells were seeded in 24 well plates at a logarithmic growth phase. The transfection was performed using a specific nanoparticle (Gift from Prof. M. Ramezani, Pharmaceutical Research Center, Mashhad University of Medical Sciences, Mashhad, Iran). The efficacy of transfection was assessed after 48 hr (Optica, Italy).

***RNA extraction, cDNA synthesis, and real-time PCR***

The paraffin-embedded tissues were treated with ethanol 96% following xylene to eliminate the paraffin. Total RNA was also isolated from ATC cell lines and paraffin-embedded tissues, using a Total RNA extraction Kit (Parstous, Iran). The cDNA synthesis was performed for miR-506 (Anacell, Iran) and mRNAs (EURX, Hungary). Quantitative polymerase chain reaction (RT-qPCR) was done by the SYBR green method (Amplicon, Denmark) in duplicate reactions (Light Cycler, Roche, Germany). Glyceraldehyde 3-phosphate dehydrogenase (GAPDH) was used as a normalizer for WNT, NOTCH, and EMT-related mRNA gene expressions, while U6 was used as a normalizer for miR-506 expression. We also assessed the levels of miR-506 expressions in paraffin-embedded ATC tissues compared with normal margins. All of the primer sequences are mentioned in Table 1. Gene expression was analyzed using the -ΔΔCT algorithm. More than a two-fold difference in fluorescence intensity was considered as up-regulation and less than two-fold indicated down-regulation in transfected cells compared with control cells. 

***Migration assay (Scratch assay)***

The 8305c cells were seeded in a 12-well plate and cultured until 90–100% confluency. Monolayer 8305c cells were scratched and washed with PBS to eliminate the detached cells. Wound closure was assessed under an inverted microscope and images were captured for measurement of scratch width at 24 hr (Optica, Italy). The percentage of wound closure was calculated by Image J software. All of the cell migration assays were performed in duplicates and significant changes were also evaluated by the ANOVA test between transfected and non-transfected cells (*P*≤0.05). 

***MTT assay (Cell viability assay)***

MTT assay was used to assess the drug response toward Cisplatin (CDDP), Paclitaxel (TXL), and Doxorubicin following the miR-506 ectopic expression in 8305c cells. MTT assay was done in 8 replicates. The cells were exposed to different concentrations of drugs in 48 hr to find half maximal inhibitory concentration (IC_50_): cisplatin (5, 10, 20, 30, 40, 80, and 160 µM), paclitaxel (1, 1.4, 3, 6, 12, 24, and 48 µM), and doxorubicin (1, 2, 4, 6, 8, 10, and 12 µM). After calculating the IC_50_ the cell viability was compared between transfected and non-transfected (control) cells. The OD of viable cells was quantified at an absorbance of 570 nm. The experiment was repeated three times.

***Cell cycle and apoptosis assays***

The 8305c cells were cultured and transfected in 6 wells plates. Then, 8305c cells were trypsinized and mixed with 70% ethanol and incubated at 4 °C for 30 min. The cells were washed and mixed with 20 µl of propidium iodide (PI) (1 mg/ml). Finally, a suspension of 500000 cells was prepared to assess cell cycle progression using flow cytometry. Apoptosis assay was performed using FITC Annexin V Kit (MabTag, GmbH, Germany). The 8305c cells were transfected with miR-506 ectopic expression plasmid and were trypsinized after 48 hr and washed with PBS. Then the cells were suspended in 90 µl of Annexin-V binding buffer 1x, 5 µl of Annexin-V conjugate, and 5 µl of PI and incubated for 20 min in darkness. Then the solution was centrifuged and the cell pellet was solved in 500 µl Annexin V binding buffer 1x. Finally, the apoptosis rate was detected using flow cytometry. The cell cycle and apoptosis were analyzed using FlowJo software. 

## Results

***Expression level of miR-506 in thyroid cancer cells and tissues***

**Figure 1:**
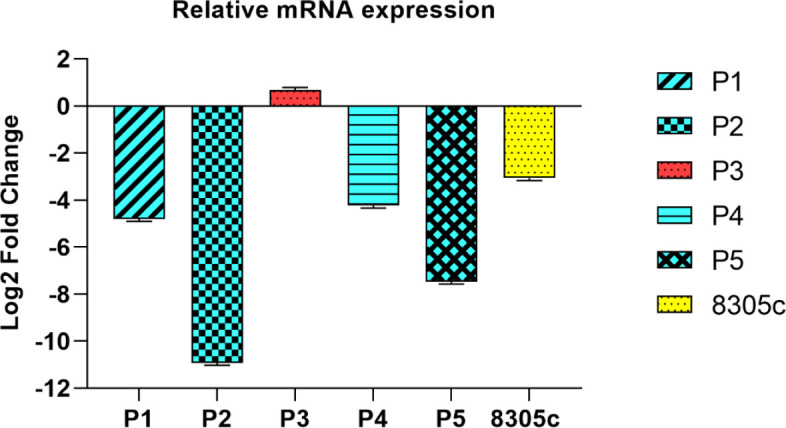
Levels of miR-506 expressions in thyroid tumor cells and clinical samples. There were significant miR-506 down-regulations in 8305c cells and the majority of the clinical samples compared with normal cells and margins

In this study, we examined the expression level of miR-506 in tumor cells compared with normal cells. The results showed that miR-506 was significantly down-regulated in 8305c cells compared with normal cells (-3.07 fold change). We also evaluated the levels of miR-506 expressions in five formalin-fixed paraffin-embedded ATC tissues compared with normal margins. There were significant miR-506 down-regulations in 4 out of 5 ATC tissues (-4.81, -10.93, 0.68, -4.24, and -7.47 fold changes) (Figure 1).

***Expression analysis of NOTCH and WNT signaling pathways***

**Figure 2.**
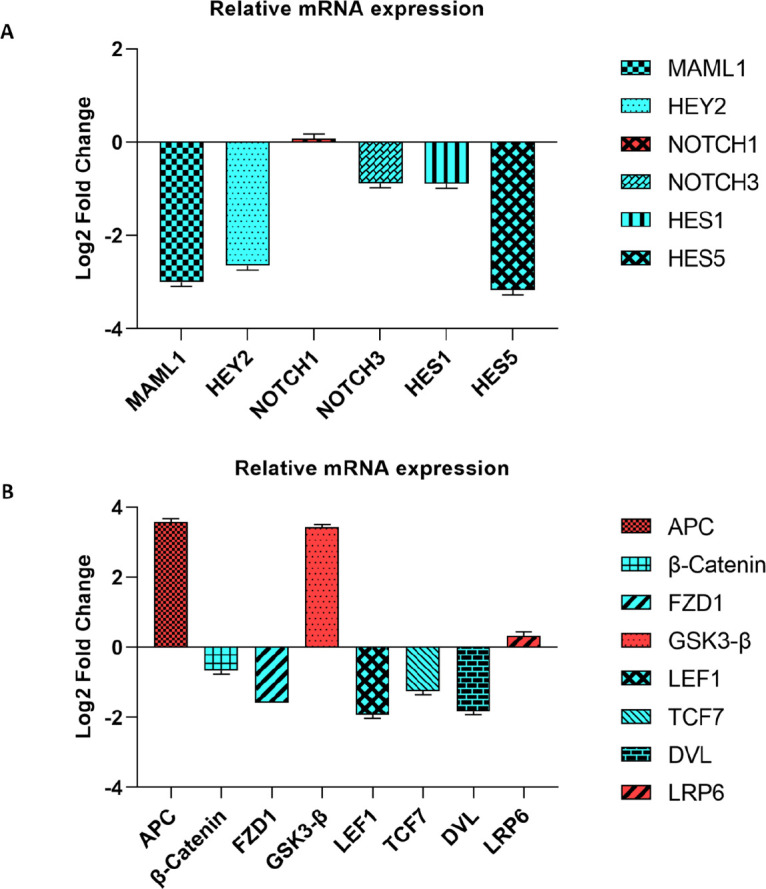
Expression analysis of NOTCH (a) and WNT (b) signaling pathways. MiR-506 suppressed NOTCH and WNT signaling pathways through LEF1, DVL, FZD1, HEY2, HES5, and HEY2 down-regulations, while APC and GSK3b were up-regulated in ATC cells

The online miRDB database predicted that miR-506 could be a direct regulator of WNT and NOTCH pathways and EMT process by targeting the FZD4, TCF3, AXIN1, LRP6, DLL4, and VIM messenger RNAs. Therefore, NOTCH and Wnt-related genes were assessed to find the molecular mechanisms that are recruited by miR-506 in the regulation of ATC cell migration and drug resistance. We considered the probable effect of miR-506 on the NOTCH signaling pathway by relative expression analysis of NOTCH-related genes between control and transfected (miR-506 ectopic expressed) cells. The relative expression of MAML1 as the main component of transcriptional machinery in the NOTCH signaling pathway was calculated, which showed a significant down-regulation in transfected cells compared with control cells (-2.99 fold changes). The relative expressions of NOTCH1 and NOTCH3 as the cell surface receptors were also calculated and there were not any significant differences between transfected and control cells (0.08 and -0.88 fold changes, respectively). The relative expressions of HEY2, HES1, and HES5 as the NOTCH target genes were also calculated (-2.64, -0.89, and -3.17 fold changes, respectively) (Figure 2a). 

Expression analysis of Wnt pathway components was also compared between transfected and non-transfected cells. There were significant APC and GSK3-B up-regulations in transfected cells compared with controls (3.58 and 3.42 fold changes, respectively). The expression levels of FZD1 and LRP6 as receptor and receptor-related proteins of the WNT pathway and other factors including β-Catenin, LEF-1, TCF-7, and DVL were also assessed between the control and transfected cells (-1.59, 0.34, -0.67, -1.93, -1.25, and -1.83 fold changes, respectively) (Figure 2). Although, there was not any significant over or under expression, we observed noticeable down-regulations in LEF-1 and DVL (Figure 2b). 

***Expression analysis of EMT and drug resistance factors***

**Figure 3:**
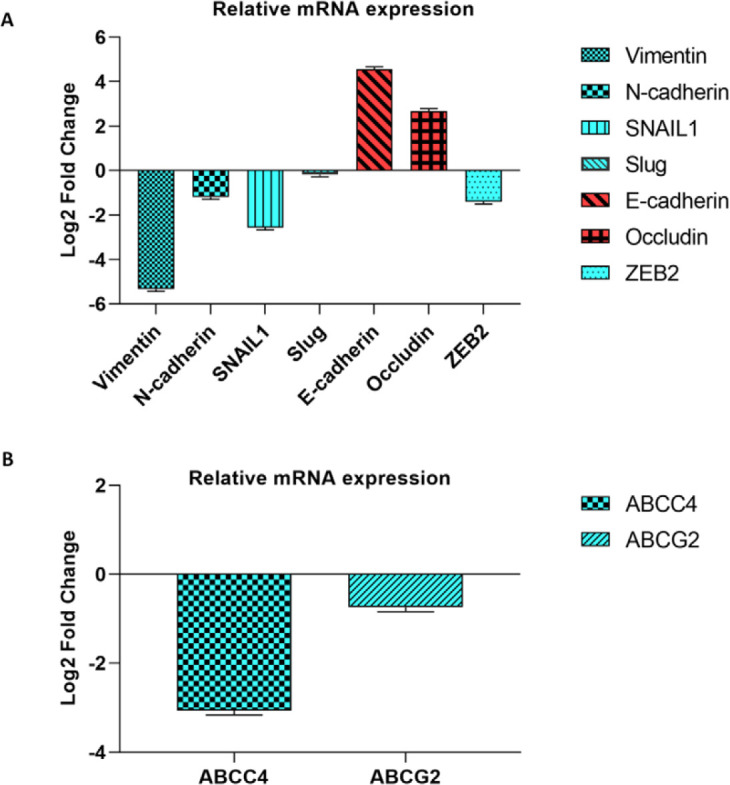
Expression analysis of EMT (a) and drug resistance factors (b). It was also observed that miR-506 significantly inhibited cell migration and EMT through VIM, SANI1, and ZEB2 down-regulations, while CDH1 and OCCLUDIN were up-regulated. Moreover, miR-506 significantly increased drug sensitivities via ABCC4 targeting in ATC cells

Tumor cell migration and metastasis via EMT are important features of aggressive tumors. In this study, we considered the effect of miR-506 on EMT factors. There were significant VIMENTIN and SNAIL under expressions (-5.34 and -2.57 fold changes, respectively), while there were significant CDH1 and OCCLUDIN up-regulations (4.56- and 2.69-fold changes, respectively) in miR-506 ectopic expressed 8305c cells compared with controls. The expression of three other EMT-related genes including N-CADHERIN, SLUG, and ZEB-2 was also considered (-1.2, -0.19, and -1.41 fold changes) (Figure 3a). Tumor recurrence is a challenging feature in the treatment of ATC that is associated with drug resistance. The effect of miR-506 on relative expressions of ABCC4 and ABCG2, as two important ABC transporters involved in drug resistance, was also compared between miR-506 transfected and non-transfected cells. There was a significant ABCC4 down-regulation in transfected cells compared with controls (-3.06 fold changes) (Figure 3b). 

***Effect of miR-506 on drug resistance and cell migration***

**Figure 4:**
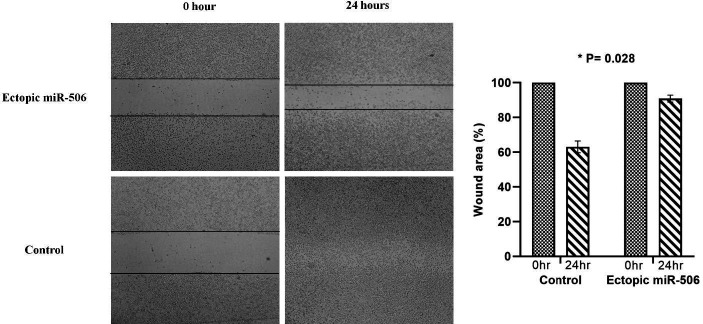
Migration assay. There was a significantly reduced cell migration in miR-506 ectopic expressed ATC cells compared with controls. The cells were monitored for 24 hr (×10 objective; OPTIKA, Italy)

**Figure 5:**
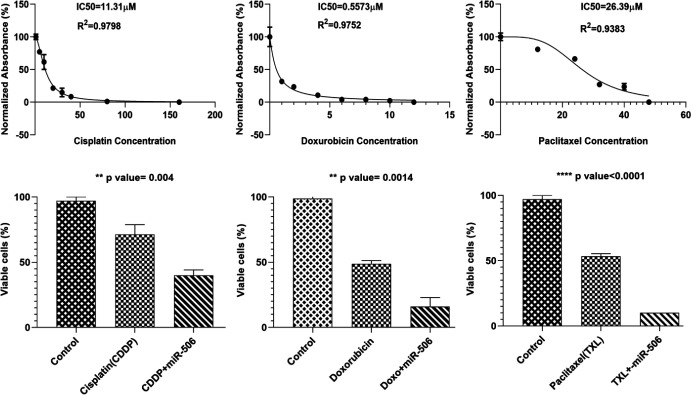
Drug resistance assay. Resistance toward Cisplatin, Paclitaxel, and Doxorubicin was significantly decreased in miR-506 ectopic expressed ATC cells compared with control cells

The effect of miR-506 on drug resistance toward Cisplatin (CDDP), Paclitaxel (TXL), and Doxorubicin was assessed after a 48 hr drug exposure. The results of the MTT assay showed that resistance toward Cisplatin, Paclitaxel, and Doxorubicin was significantly decreased in miR-506 transfected cells compared with control cells (*P*=0.004, *P*<0.0001, and *P*=0.0014, respectively) (Figure 4). The rate of cell migration was also assessed via scratch assay after 24 hr between the miR-506 transfected cells and non-transfected cells. MiR-506 significantly decreased 8305c cell migration in transfected cells compared with controls (*P*=0.028) (Figure 5).

***Effect of miR-506 on cell cycle and apoptosis***

**Figure 6:**
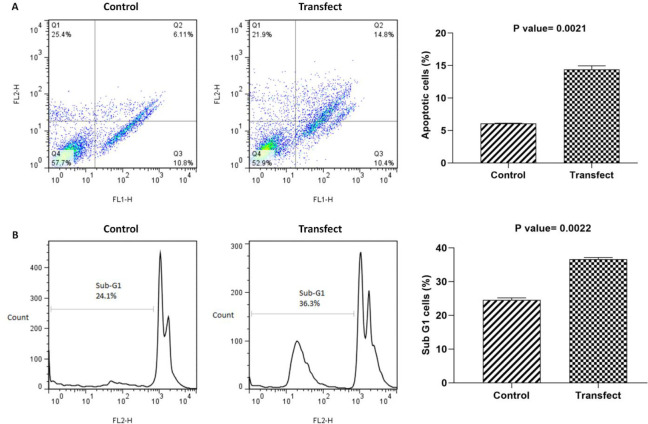
Cell cycle and apoptosis assays. There was a significantly increased percentage of apoptotic cells in transfected cells compared with control cells (a). The percentage of cells in the sub-G1 phase was also significantly increased in transfected cells compared with non-transfected cells (b)

**Figure 7:**
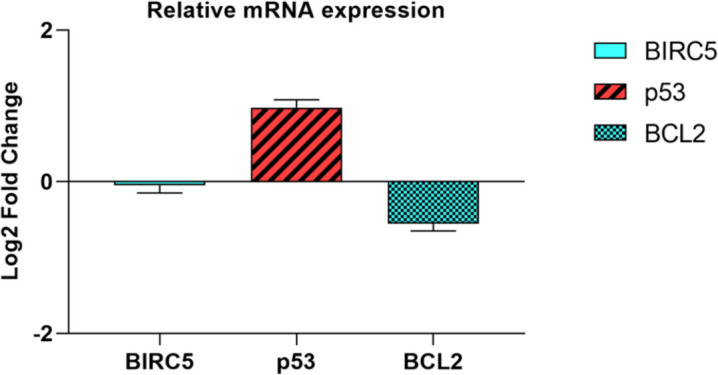
Expression levels of apoptosis-related genes were also assessed in transfected cells compared with controls. There were not any significant changes in the levels of apoptosis-related gene expressions following the miR-506 ectopic expression in ATC cells

Cell cycle and apoptosis assays were performed using the Annexin/PI flow cytometry to compare the percentage of sub-G1 and apoptotic cells between the control and transfected cells. There was a significantly increased percentage of apoptotic cells in transfected cells compared with control cells (14.8% vs 6.11%) (*P*=0.0021) (Figure 6a). The percentage of cells in the sub-G1 phase was also significantly increased in transfected cells compared with non-transfected cells (36.3% vs 24.1%) (*P*=0.0022) (Figure 6b). In addition to apoptosis assay, the relative expression of P53, Birc5, and BCL2 was also assessed in transfected cells compared with controls (0.98, -0.05, and -0.55 fold changes, respectively) (Figure 7).

## Discussion

ATC is a malignant thyroid tumor type that has a poor prognosis due to its high therapeutic resistance. Although ATC is a rare type of thyroid tumor that accounts for 1-2% of cases, it is responsible for up to 50% of thyroid tumor-related mortalities. There is not still an efficient therapeutic modality for locally advanced or metastatic ATC tumors. Therefore, it is required to clarify the molecular mechanisms of thyroid cancer progression and metastasis to introduce novel diagnostic and therapeutic methods. MiRNAs are pivotal factors during thyroid cancer initiation and progression (29). MiR-506 has mainly a tumor suppressor function in various tumors such as cervical and breast cancers (30, 31). It has been reported that miR-506-3p significantly reduced PTC cell proliferation by inhibition of the YAP1-CDK2/CCNE1 axis (32). MiR-506 reduced PTC cell proliferation and invasion through IL17RD sponging (33). However, the molecular mechanisms of miR-506 in ATC drug response have not been investigated. In this study, we assessed the potential role of Wnt and NOTCH signaling pathways in cell migration and chemotherapeutic responses in 8305c cells. We first evaluated the miR-506 expression levels in five paired clinical anaplastic tumor tissues and their normal margins, and observed that there were significantly reduced levels of miR-506 expressions in the majority of ATC samples. We also showed that the 8305c cells had lower levels of miR-506 expression compared with normal thyroid tissues. 

MicroRNAs have pivotal roles in drug resistance through regulation of cell cycle progression, apoptosis, drug transport, DNA repair, and EMT. Platinum-based compounds form DNA adducts that promote DNA repair mechanisms following the DNA double-strand breaks. It has been reported that miR-506 increased cisplatin response via RAD51 targeting (34). MiR-506-3p increased olaparib and cisplatin sensitivities by β-catenin targeting in ovarian tumor cells (35). It was found that miR-506 repressed pancreatic tumor cell growth while increasing apoptosis and chemosensitivity by SPHK1/Akt/NF-κB axis (36). MiR-506 targeted the EZH2 and Wnt/β-catenin signaling pathway to increase sensitivity toward PARP inhibitors and cisplatin in ovarian tumor cells (35). ABC transporters are important factors in drug efflux and their dysfunction has an important role in treatment failure. MiR-506 down-regulation has been observed in chemo-resistant colorectal tumors. MiR-506 down-regulated the MDR1/P-gp through Wnt/β-catenin pathway inhibition to reverse oxaliplatin resistance (37). MiR-506 was also involved in the chemo-response of ovarian and colorectal tumors via RAD51 and PPARα, respectively (28, 38). ABCC4 belongs to the ABC protein family that transports different organic anions (39). It has been shown that there was significant miR-506 down-regulation in cervical cancer (CC) tissues compared with normal margins. MiR-506 suppressed CC cell proliferation via ABCC4 targeting (40). In another study, miR-506 and miR-124a targeted ABCC4 (41). We also observed that miR-506 increased cisplatin, doxorubicin, and paclitaxel sensitivity through ABCC4 down-regulation in anaplastic thyroid tumor cells. 

EMT is a cellular process that allows the epithelial cells to lose their adhesion and obtain the mesenchymal features, resulting in increased cell motility and invasion. EMT is usually orchestrated by down-regulation of epithelial factors including CDH1 and OCCLUDIN, while up-regulatimg mesenchymal factors such as CDH2 and VIM (42). MiR-506 functions as a tumor suppressor by EMT suppression (43). There was a converse correlation between the levels of miR-506 and SNAI2 expressions in gastric tumor tissues. There were also miR-506 and CDH1 up-regulations while SNAI2 down-regulation in gastric tumor cells resulted in reduced cell migration (44). It has been observed that FOXD2-AS1 long non-coding RNA (lncRNA) induced glioma cell proliferation and EMT by CDH1, CDH2, and VIM regulations through miR-506-5p sponging (45). HOXA11AS promoted hepatocellular carcinoma cell proliferation and EMT through miR5063p/Slug axis in hepatocellular carcinoma (46). EMT is also involved in the chemoresistance of tumor cells (47). NEAT1 regulates ZEB2 expression by miR-506-3p targeting that results in increased gemcitabine resistance in pancreatic tumor cells through miR-506- 3p/ZEB2/EMT axis (48). In another study on gastric cancer, miR-506 reduced cell migration and invasion via ZEB2 sponging (49). We observed that miR-506 reduced anaplastic thyroid tumor cell migration and EMT process through significant CDH1 and OCCLUDIN up-regulations, and SNAI1 and VIMENTIN down-regulations. There were also insignificant CDH2 and ZEB2 down-regulations following the miR-506 ectopic expression in ATC cells. 

**Figure 8:**
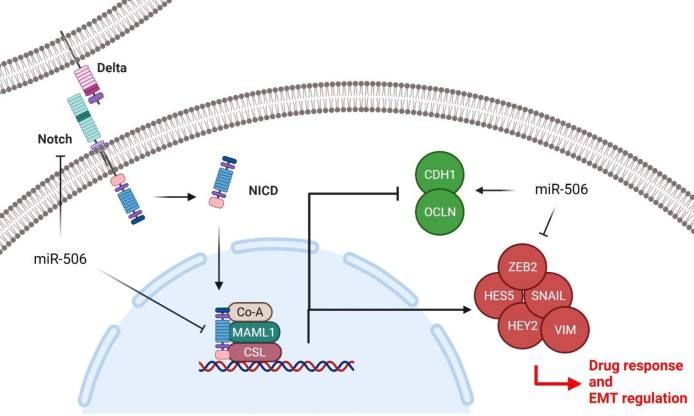
Role of miR-506 in regulation of NOTCH and EMT processes in ATC cells

NOTCH signaling is involved in various cellular processes such as cell proliferation, migration, and tumorigenesis. It is a cell-cell contact-related pathway that is triggered by binding of the NOTCH ligands (Jagged 1-2 and Delta 1/3/4) to the NOTCH receptors (NOTCH1-4), leading to the production of NOTCH intracellular domain (NICD). Subsequently, NICD translocates into the nucleus where it binds with CSL/MAML1 transcriptional complex to regulate the NOTCH target genes such as HES and HEY (12). Various miRNAs are involved in the regulation of the NOTCH signaling pathway in thyroid tumor cells (50). It has been observed that miR-449 suppressed NOTCH signaling via NOTCH1 sponging which reduced PTC cell proliferation and migration while inducing apoptosis (51). MiR-182 has an oncogenic role in medullary thyroid carcinoma through regulation of the HES1/NOTCH1 axis (52). There are also some reports about the role of miR-506 in the regulation of NOTCH signaling in tumor cells. LINC01410 up-regulated NOTCH2 and activated NOTCH signaling by miR-506-3p sponging in glioma cells (53). MiRNAs can also affect the drug responses in tumor cells by regulating NOTCH signaling. It has been reported that miR-139-5p increased 5-FU sensitivity by NOTCH-1 targeting in colorectal tumor cells (54). We also assessed the probable role of miR-506 in the regulation of the NOTCH signaling pathway in ATC cells for the first time. Interestingly, we observed that miR-506 suppressed NOTCH signaling via down-regulation of MAML1 as the main co-transcription factor of the NOTCH pathway and some of the NOTCH target genes such as HEY2 and HES5. However, we observed that there was not any correlation between the miR-506 and NOTCH receptors such as NOTCH1 and NOTCH3 in ATC cells (Figure 8). 

**Figure 9:**
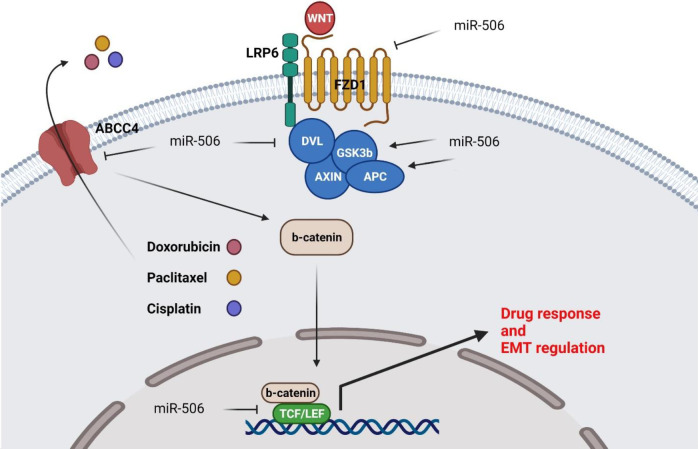
Role of miR-506 in regulation of WNT and EMT processes in ATC cells

Wnt/β-catenin is also a developmental signaling pathway involved in cell proliferation, invasion, and apoptosis (55). There is a correlation between miR-506 and WNT signaling in the regulation of drug response in tumor cells. It has been shown that miR-506-3p reduced cisplatin resistance via CircUBAP2/SEMA6D/miR-506-3p axis that affects the Wnt signaling in osteosarcoma (OS) cells (56). MiR-506 significantly reduced colorectal tumor cell proliferation and migration by EZH2 targeting and WNT signaling regulation (57). We assessed the role of miR-506 in the regulation of the WNT signaling pathway in ATC cells. It was observed that miR-506 suppressed WNT signaling by APC and GSK3b up-regulations, while LEF1, FZD1, DVL, and TCF7 were down-regulated in miR-506 ectopic expressed 8305c cells compared with control cells. Regarding our results, miR-506 mainly suppressed WNT by regulation of the cytoplasmic destruction complex (Figure 9). 

In a study on Mantle cell lymphoma tissues and cell lines, miR-506 induced cell cycle arrest at G0/G1 by targeting B7H3 (58). MiR-506 also induced cell cycle arrest by targeting MTMR6 in ovarian tumor cells (59). We also assessed the role of miR-506 in cell cycle progression and apoptosis in ATC cells. We observed that there was a significantly increased percentage of apoptotic and sub-G1 cells in miR-506 ectopic expressed 8305c cells compared with control cells. However, we did not observe any significant changes in the levels of apoptosis-related genes such as BIRC5, p53, and BCL2 following miR-506 ectopic expression. 

***Limitations***

Animal studies following the miR-506 transfection are required to clearly conclude the tumor suppressive role of this miRNA in ATC cells. Assessment of circulating miR-506 levels is required in a higher number of ATC patients to introduce that as an efficient non-invasive marker in ATC patients. In the present study, we only assessed the levels of mRNA expressions in miR-506 target genes. However, protein studies of the WNT, NOTCH, and EMT pathways are also recommended to clarify the role of miR-506 in regulation of such pathways in ATC cells. 

**Table 1:** Primer sequences for the real-time PCR

Gene	Primer sequences	Thermal profile	Size (bp)
MAML1	F= TCTCGCGGAACAGGAGAA R=GCAGCAGAGGACCCTGTG	95ͦ C(15 min)[95ͦ C (15 s)/60ͦ C (30 s)/72ͦ C (30 s)]45	123
HEY2	F= ATGAGCATAGGATTCCGAGAGTG R= GGCAGGAGGCACTTCTGAAG	95ͦ C(15 min)[95ͦ C (15 s)/60ͦ C (30 s)/72ͦ C (30 s)]45	300
NOTCH1	F= CAGAGGCGTGGCAGACTAT R= CGGCACTTGTACTCCGTCAG	95ͦ C(15 min)[95ͦ C (15 s)/604ͦ C (30 s)/72ͦ C (30 s)]45	147
NOTCH3	F= AGGGACGTCAGTGTGAACTC R= GTCCACATCCTGCTGGCATC	95ͦ C(15 min)[95ͦ C (15 s)/602ͦ C (30 s)/72ͦ C (30 s)]45	143
HES1	F=GGCTAAGGTGTTTGGAGGCT R=GCTGTTGCTGGTGTAGACGG	95ͦ C(15 min)[95ͦ C (15 s)/57.5ͦ C (30 s)/72ͦ C (30 s)]45	121
APC	F= ACGAGCACAGCGAAGAATAGC R=TGTAGTTGAACCCTGACCATTACC	95 C(15 min)[95ͦ C (15 s)/60ͦ C (30 s)/72ͦ C (30 s)]45	211
Catenin	F=CAACTAAACAGGAAGGGATGGAAGG R= CAGATGACGAAGAGCACAGATGG	95ͦ C(15 min)[95ͦ C (15 s)/57.5ͦ C (30 s s)/72ͦ C (30 s)]45	239
FZD-1	F=CAAGAGAGGAGCCGAGAAAGTATG R=CCAGCAGCCAAAGCAGCAG	95 ͦC(15 min)[95ͦ C (15 s)/57ͦ C (15 s)/72ͦ C (15s)]45	208
GSK3-B	F=AGTGGTGAGAAGAAAGATGAGGTC R=GTTTAATATCCCGATGGCAGATTCC	95 ͦC(15 min)[95ͦ C (15 s)/60.5ͦ C (15 s)/72ͦ C (15s)]45	196
LEF-1	F=CAGCGGAGCGGAGATTACAG R=GATTTCAGACTCGTTCACCAAGG	95 ͦC(15 min)[95ͦ C (15 s)/56ͦ C (30 s)/72ͦ C (30 s)]45	219
TCF-7	F= GCTGCCATCAACCAGATCCT R= CCTCCTGTGGTGGATTCTTGG	95 ͦC(15 min)[95ͦ C (15 s)/61 ͦC (30 s)/72ͦ C (30 s)]45	191
DVL	F=GGTCTCCTGGCTGGTCCTG R=CCTGTCTCGTTGTCCATCCC	95 ͦC(15 min)[95ͦ C (15 s)/61ͦ C (30 s)/72ͦ C (30 s)]45	184
LRP6	F=AACCTTCAAGAATACAGACAGCAC R=TCTTCACATTCAGTAAACCCATCG	95 ͦC(15 min)[95ͦ C (15 s)/61ͦ C (30 s)/72ͦ C (30 s)]45	215
VIMENTIN	F=GGCTCGTCACCTTCGTGAAT R=GAGAAATCCTGCTCTCCTCGC	95ͦC(15 min)[95ͦ C (15 s)/60ͦ C (30 s)/72ͦ C (30 s)]45	110
N-CADHERIN	F=ATGGTGTATGCCGTGAGAAG R=TGTGCTTACTGAATTGTCTTGG	95ͦ C(15 min)[95ͦ C (15 s)/61ͦ C (30 s)/72 ͦC (30 s)]45	196
SNAIL	F=CTAGGCCCTGGCTGCTACAA R=ACATTCGGGAGAAGGTCCGA	95 ͦC(15 min)[95ͦ C (15 s)/60 ͦC (30 s)/72ͦ C (30 s)]45	177
SLUG	F=GCCAAACTACAGCGAACTGG R=TGGAATGGAGCAGCGGTAG	95 ͦC(15 min)[95 ͦC (15 s)/60 ͦC (30 s)/72 ͦC (30 s)]45	150
E-CADHERIN	F=ATTCTGATTCTGCTGCTCTTG R=AGTCCTGGTCCTCTTCTCC	95 ͦC(15 min)[95ͦ C (15 s)/60ͦ C (30 s)/72ͦ C (30 s)]45	136
ZEB-2	F=GGGACAGATCAGCACCAAAT R=CGCAGGTGTTCTTTCAGATG	95 ͦC(15 min)[95ͦ C (15 s)/59ͦ C (30 s)/72ͦ C (30 s)]45	204
OCCLUDIN	F=AAGCAAGTGAAGGGATCTGC R=GGGGTTATGGTCCAAAGTCA	95 ͦC(15 min)[95ͦ C (15 s)/59ͦ C (30 s)/72ͦ C (30 s)]45	110
ABCC4	F=GAAATTGGACTTCACGATTTAAGG R=TTCCACAGTTCCTCATCCGT	95 ͦC(15 min)[95ͦ C (15 s)/60ͦ C (30 s)/72ͦ C (30 s)]45	125
ABCG2	F=TGAGGGTTTGGAACTGTGG R=GATTCTGACGCACACCTGG	95 ͦC(15 min)[95ͦ C (15 s)/56ͦ C (30 s)/72ͦ C (30 s)]45	155

EMT: Epithelial-mesenchymal transition; ATC: Anaplastic thyroid carcinoma

## Conclusion

We have recently reported that the WNT and NOTCH signaling pathways are pivotal regulators of EMT, cell migration, and drug responses in tumor cells (12, 60). Therefore, miR-506 can also affect EMT, cell migration, and chemoresistance through regulation of WNT and NOTCH signaling pathways in ATC cells. MiR-506 can be suggested as an efficient novel therapeutic factor for ATC tumors. However, further animal studies are required to confirm miR-506 as a therapeutic target in clinics. Assessment of the circulating miR-506 is also required in a higher number of ATC patients to efficiently suggest it as a reliable non-invasive tumor marker in ATC patients. 

## Authors’ Contributions

ZNN, NT, ASZ, and MRA were involved in experiments, drafting, and edition. MM analyzed data and supervised the project. All authors read and approved the final manuscript.

## Ethics Approval

All the participants filled out informed consent forms that were approved by the ethics committee of Mashhad University of Medical Sciences. The study was performed in accordance with the ethical standards as laid down in the 1964 Declaration of Helsinki and its later amendments or comparable ethical standards.

## Consent to Participate and Publish

Written informed consent was obtained from all participants to participate in the study and also to have their data published without any identifying information.

## Conflicts of Interest

The authors declare that they have no competing interests.
